# Elimination of Neglected Diseases in Latin America and the Caribbean: A Mapping of Selected Diseases

**DOI:** 10.1371/journal.pntd.0000964

**Published:** 2011-02-15

**Authors:** Maria Cristina Schneider, Ximena Paz Aguilera, Jarbas Barbosa da Silva Junior, Steven Kenyon Ault, Patricia Najera, Julio Martinez, Raquel Requejo, Ruben Santiago Nicholls, Zaida Yadon, Juan Carlos Silva, Luis Fernando Leanes, Mirta Roses Periago

**Affiliations:** Pan American Health Organization, Washington, D.C., United States of America; London School of Hygiene & Tropical Medicine, United Kingdom

## Abstract

In Latin America and the Caribbean, around 195 million people live in poverty, a situation that increases the burden of some infectious diseases. Neglected diseases, in particular, are often restricted to poor, marginalized sections of the population. Tools exist to combat these diseases, making it imperative to work towards their elimination. In 2009, the Pan American Health Organization (PAHO) received a mandate to support the countries in the Region in eliminating neglected diseases and other poverty-related infections. The objective of this study is to analyze the presence of selected diseases using geo-processing techniques. Five diseases with information available at the first sub-national level (states) were mapped, showing the presence of the disease (“hotspots”) and overlap of diseases (“major hotspots”). In the 45 countries/territories (approximately 570 states) of the Region, there is: lymphatic filariasis in four countries (29 states), onchocerciasis in six countries (25 states), schistosomiasis in four countries (39 states), trachoma in three countries (29 states), and human rabies transmitted by dogs in ten countries (20 states). Of the 108 states with one or more of the selected diseases, 36 states present the diseases in overlapping areas (“major hotspots”). Additional information about soil-transmitted helminths was included. The analysis suggests a majority of the selected diseases are not widespread and can be considered part of an unfinished agenda with elimination as a goal. Integrated plans and a comprehensive approach, ensuring access to existing diagnostic and treatment methods, and establishing a multi-sectoral agenda that addresses social determinants, including access to adequate water and sanitation, are required. Future studies can include additional diseases, socio-economic and environmental variables.

## Author Summary

Neglected diseases are often restricted to poor, marginalized sections of the population. Tools exist to combat these diseases, making it imperative to work towards their elimination. In 2009, the Pan American Health Organization received a mandate to support the countries in the Region in eliminating neglected diseases and other poverty-related infections. This study analyzed the presence of selected diseases using geo-processing techniques. Five diseases (lymphatic filariasis, onchocerciasis, schistosomiasis, trachoma and human rabies transmitted by dogs) were mapped by state, showing the presence of the disease (“hotspots”) and overlap of diseases (“major hotspots”). Additional information about soil-transmitted helminths was included. In the 45 countries/territories (approximately 570 states) of the Region, there are 108 states with one or more of the selected diseases, and 36 states present the diseases in overlapping areas (“major hotspots”). Integrated plans and a comprehensive approach, ensuring access to existing diagnostic and treatment methods, and establishing a multi-sectoral agenda that addresses social determinants, are required.

## Introduction

Of the 580 million people who live in Latin America and the Caribbean, around 195 million live in poverty (defined as earning less than two dollars a day), and 71 million live in extreme poverty (defined as earning less than one dollar per day) [1]. Most of them, including indigenous populations, rural poor, slum residents, migrant workers, the elderly, and women and children, live in unfavorable conditions and suffer a greater burden of infectious diseases [2].

The higher burden of parasitic and other diseases could be related to insufficient access to drinking water, sanitation, inadequate housing, education, and health services [3], [4]. Most of these diseases cause chronic conditions that can reduce learning capabilities, productivity, and income earning capacity. Thus, these neglected diseases are both a cause and a consequence of poverty [5].

The availability of new tools, the improvement of health service infrastructure, and the implementation of new strategies—particularly rising emphasis on primary care—make it feasible to control and eliminate selected diseases. The elimination and eradication of diseases has been the topic of several conferences, workshops, and publications over the past two decades [6]–[9]. Some of the diseases listed as potential targets for elimination are also included in World Health Organization's (WHO) 2008–2015 Global Plan to Combat Neglected Tropical Diseases such as onchocerciasis and lymphatic filariasis [2].

The Region of the Americas has successfully implemented elimination and eradication strategies for diseases such as smallpox, polio, and measles [10]. The countries have also expressed political commitment to addressing poverty-related disease elimination. They have approved various WHO and PAHO resolutions on neglected diseases such as Chagas Disease, onchocerciasis, congenital syphilis, leprosy and others [11]–[17].

Recently, PAHO's Directing Council approved a resolution on “Elimination of Neglected Diseases and other Poverty-related Infections” which reinforced the countries' and the Organization's commitment to combating diseases affecting neglected populations using integrated plans and comprehensive approaches [18].

This resolution identified twelve target diseases, which were selected by several criteria such as being part of the unfinished agenda, technical feasibility, and regional evidence of achievable elimination among others. The selected diseases were divided into two groups–those with greater potential for being eliminated, and those that can be drastically reduced with available tools. Group 1 includes diseases that have a greater potential for being eliminated: ‘Chagas’ disease (vector-borne and transfusional transmission, both as a public health problem); congenital syphilis (as a public health problem); lymphatic filariasis (as a public health problem); onchocerciasis; rabies transmitted by dogs; neonatal tetanus (as a public health problem); trachoma (as a public health problem); leprosy (as a public health problem at the national and first subnational level); malaria (elimination in Haiti and the Dominican Republic and in Mexico and Central America); plague (as a public health problem). Group 2 encompasses diseases whose burden can be drastically reduced with available tools: schistosomiasis and soil-transmitted helminthiasis. Elimination was defined in this Resolution as the reduction to zero of the incidence of a given disease in a defined geographic area as a result of deliberate efforts, with continued intervention measures being required, while elimination of a disease as a public health problem was defined as drastically reducing the disease's burden to a level that is acceptable given the current tools available and the health situation, such that, the prevalence of the disease does not constrain social productivity nor community development. Achievable goals were established for each disease [18].

Among the twelve selected diseases, five have disaggregated data available for all countries/territories of Latin America and the Caribbean and were therefore the focus of this study, complemented by a sixth disease with partial information.

The purpose of this paper is to present a set of epidemiological information available on the geographic presence of the selected diseases to support decision makers in their efforts to meet the commitment of eliminating these diseases from the Region. This analysis allows for the identification of the presence of the diseases at the country and first sub-national levels, visualizing areas where they overlap, that can be used in interprogrammatic and intersectoral approaches, as well as taking into account potential for cross-border activities. No effort has been conducted in the past to map the presence and overlap of these diseases, using standardized indicators, for all of Latin America and the Caribbean.

## Methods

### Selected diseases and data

Five of the twelve diseases in the PAHO resolution cited above – lymphatic filariasis, onchocerciasis, human rabies transmitted by dogs, schistosomiasis, and trachoma – had data available disaggregated at the first sub-national level at the time of this study. These five diseases are reviewed in this article including individual maps presenting the critical areas for intervention (“hotspots”) by disease and the overlapping of disease maps in order to identify “major hotspots” as targets for possible interprogrammatic and intersectoral interventions by the countries. Various definitions of “hotspot” were reviewed to create the definitions used in this study [19]–[21]. As the diseases in this article have already been targeted for elimination, any presence is considered a “hotspot” while overlapping presence is considered even more critical as a “major hotspot.”

There are 45 countries and territories in Latin America and the Caribbean, with around 570 first sub-national level units within the countries. These could be a state, department, or province according to the administrative geopolitical units in the countries and territories. All countries/territories and states in the Region of the Americas are included in this analysis.

Only secondary sources of information were used, primarily information that has been published or uploaded to the websites of the respective Ministries of Health or of organizations that have mandates for technical cooperation on the subject (PAHO, WHO, and the Onchocerciasis Elimination Program for the Americas, OEPA). For diseases for which this information was not available on the official websites, other sources of information were used, such as publications, reports from Regional or sub-regional plans, and presentations from technical/scientific meetings organized by PAHO/WHO, which were provided by the PAHO Regional Advisors on the subjects. A preliminary document was prepared with the same information used in this study and was uploaded to PAHO's website, allowing the countries to send in comments and validate the included information [22]. This document, with over 100 references, is used as the primary source of information for the analysis conducted in this paper.

For rabies, onchocerciasis, and lymphatic filariasis, the criteria used was evidence of the presence of the disease over a three year period (2005 to 2007) because the epidemiological situation may vary in a short period of time and elimination programs are in place with updated information for most areas. For trachoma and schistosomiasis, the epidemiological situation usually does not vary in short periods and there is a lack of periodic information for many countries; the presence of the disease is based on surveys conducted over a longer interval of years. For this reason, the criterion used was evidence of the presence of the disease over the 10 year period between 1998 to 2007. A database was created with the information obtained at the first sub-national level and this data was utilized for geo-processing.

In the case of the soil-transmitted helminthiases (STH) *Ascaris lumbricoides*, *Trichuris trichiura* and hookworms *(Ancylostoma duodenale*, and *Necator americanus*), which were targeted for drastic reduction by the Resolution, information was not available for all countries/territories but only for some geopolitical units. Given these circumstances and their public health importance, a separate analysis was carried out. PAHO did an extensive literature search to compile published data on prevalence and intensity of infection throughout the Region during the period 1998 to 2007, and built a database with data on prevalence of infection which included information on 526 studies. However, most of the studies were localized point prevalence studies covering a broad range in the number of people surveyed, in the target age groups for the studies, and with a great heterogeneity in the geographical units targeted, as well as in the epidemiological and laboratory methods for assessing prevalence of infection. To minimize the statistical errors, the 95% confidence intervals for all of the prevalence studies found were calculated. Given this information, states were divided into those with a prevalence equal to or greater than 20% in school-age children and those below that threshold.

### Cartography and geo-processing

The cartography was made available by PAHO's Health Analysis and Information Project (HSD/HA), which compiled data from a range of different sources, including the WHO Evidence, Information, and Research Project (EIR) First Administrative Level Boundaries dataset (EIP_1admin, Version 1) and the digital cartography provided by the countries' health departments and/or the national geographic agencies. HSD/HA standardized the sub-national administrative units according to WHO coding schemes [23].

For this study, several geo-processing techniques were used, including geo-coding the selected diseases' statistical databases, applying specific cartographic projections to the Regional and national levels, performing spatial queries to assemble the choropleth mapping classification schemes on the different geographic analysis units, and generating new dicothomic variables in order to identify spatial distribution patterns, as well as the geographic overlap of neglected diseases. The geo-processing and spatial analyses were performed in the Geographic Information Systems (GIS) software ArcView 3.3.

## Results

There is evidence of the presence of selected diseases analyzed in this study in a limited number of countries of the Americas and for most of these countries, the presence is limited to specific first-sub-national level units (Table 1). The information indicates that 108 (19%) of 570 first-sub-national level units have one or more of the selected neglected diseases, thus they may be considered “hotspots” in the Region or critical areas for intervention. More detailed information by disease and the overlapping “major hotspots” can be found below. In addition, the goals and primary strategies approved by PAHO's Directing Council for the selected diseases are included in Table 2 [18].

**Table 1:** Presence of selected neglected diseases, by country, Latin America and the Caribbean*.

Country and territories	Disease				
	Lymphatic filariasis	Onchocerciasis	Human Rabies transmitted by dogs	Schistosomiasis	Trachoma
Anguilla	-	-	-	-	-
Antigua and Barbuda	-	-	-	-	-
Argentina	-	-	-	-	-
Aruba	-	-	-	-	-
Bahamas	-	-	-	-	-
Barbados	-	-	-	-	-
Belize	-	-	-	-	-
Bolivia	-	-	X	-	-
Brazil	X	X	X	X	X
Cayman Islands	-	-	-	-	-
Chile	-	-	-	-	-
Colombia	-	X	X	-	-
Costa Rica	-	-	-	-	-
Cuba	-	-	X	-	-
Dominica	-	-	-	-	-
Dominican Republic	X	-	-	-	-
Ecuador	-	X	-	-	-
El Salvador	-	-	X	-	-
French Guiana	-	-	-	-	-
Grenada	-	-	-	-	-
Guadeloupe	-	-	-	-	-
Guatemala	-	X	X	-	X
Guyana	X	-	-	-	-
Haiti	X	-	X	-	-
Honduras	-	-	-	-	-
Jamaica	-	-	-	-	-
Martinique	-	-	-	-	-
Mexico	-	X	X	-	X
Montserrat	-	-	-	-	-
Netherlands Antilles	-	-	-	-	-
Nicaragua	-	-	-	-	-
Panama	-	-	-	-	-
Paraguay	-	-	-	-	-
Peru	-	-	X	-	-
Puerto Rico	-	-	-	-	-
Saint Kitts and Nevis	-	-	-	-	-
Saint Lucia	-	-	-	X	-
Saint Vincent and the Grenadines	-	-	-	-	-
Suriname	-	-	-	X	-
Trinidad and Tobago	-	-	-	-	-
Turks and Caicos Islands	-	-	-	-	-
Uruguay	-	-	-	-	-
Virgin Islands (UK)	-	-	-	-	-
Virgin Islands (USA)	-	-	-	-	-
Venezuela	-	X	X	X	-
**Total number countries with evidence of the diseases**	**4/45**	**6/45**	**10/45**	**4/45**	**3/45**
**Total number first-sub national level with evidence of the diseases**	**29/570**	**25/570**	**20/570**	**39/570**	**29/570**

- No evidence

*Criteria:

Lymphatic filariasis:Evidence of the disease in the last 3 years (2005-2007)

Onchocerciasis: Evidence of the disease in the last 3 years (2005-2007)

Rabies transmitted by dogs: Evidence of the disease in the last 3 years (2006-2008)

Schistosomiasis: Evidence of the disease in the last 10 years (1998-2007)

Trachoma: Evidence of the disease in the last 10 years (1998-2007)

PAHO/HSD/CD. Epidemiological Profiles of Neglected Diseases and Other Infections Related to Poverty in Latin America and the Caribbean. Presented at the Consultation on a Latin American and Caribbean Trust Fund for the Prevention, Control and Elimination of Neglected and Other Infectious Diseases. Washington, DC, 15-16 December 2008. Available at: http://new.paho.org/hq/index.php?option=com_joomlabook&Itemid=259&task=display&id=37.

**Table 2:** Goals and primary strategies by selected disease.

Disease	Goals	Primary strategies
**Lymphatic filariasis**	– To eliminate the disease as a public health problem (less than 1% prevalence of microfilaria in adults in sentinel sites and spot-check sites in the area).– Interrupt its transmission (no children between ages 2 and 4 are antigen-positive).– To prevent and control disability.	– Mass drug administration once a year for at least 5 years with coverage of no less than 75% or consumption of diethylcarbamazine-fortified table salt in the daily diet.– Surveillance of LF morbidity by local health surveillance systems.– Morbidity case management.– Integration/coordination of MDA with others strategies.– Communication strategies and education in schools.
**Onchocerciasis**	– To eliminate ocular morbidity and to interrupt transmission.	– Mass drug treatment administration at least twice a year in order to reach at least 85% of the eligible population in each endemic area.– Surveillance for signs of ocular morbidity, microfilaria, nodules.– Dermatological care through the primary health care system in areas where skin infection is a problem.
**Schistosomiasis**	– To reduce prevalence and parasite load in high transmission areas to less than 10% prevalence as measured by quantitative egg counts.	– Preventive chemotherapy for at least 75% of school-age children that live in at-risk areas, defined by a prevalence over 10% in school-age children.– Improvements of excreta disposal systems and access to drinking water, education.
**Human rabies transmitted by dogs**	– To eliminate human rabies transmitted by dogs (zero cases reported to the Epidemiological Surveillance System for Rabies (SIRVERA) coordinated by PAHO).	– Vaccination of 80% of the canine population in endemic areas.– Care given to 100% of the exposed population at risk with post-exposure prophylaxis when indicated.– Epidemiological surveillance.– Education and communication to increase awareness of the risk of rabies.
**Trachoma**	– To eliminate new cases of blindness caused by trachoma (reduction in the prevalence of trachomatous trichiasis to less than 1 case per 1,000 (general population) and reduction in the prevalence of follicular or inflammatory trachoma (FT and IT) to less than 5% in children aged 1-9 years).	– The “SAFE” strategy is used with the following components:• To prevent blindness through eyelid surgery to correct the inversion or entropy of the upper eyelid and trichiasis.• To reduce the transmission in endemic areas by washing of the face and by using antibiotics.
**Soil-transmitted helminths**	- To reduce prevalence among school-age children in high risk areas (prevalence >50%) to less than <20% prevalence as measured by quantitative egg count.	– Regular administration of preventive chemotherapy/or mass drug administration (MDA) for at least 75% of school-age children at risk, as defined by the countries considering the prevalence. If prevalence of any soil-transmitted helminthiasis infection among school-age children is ≥50% (high-risk community), treat all school-age children twice each year. If prevalence of any soil-transmitted helminthiasis infection among at-risk school-age children is ≥20% and <50% (low-risk community), treat all school-age children once each year.- Promoting access to safe water, sanitation and health education, through intersectoral collaboration.

### Lymphatic filariasis

**Figure 1:**
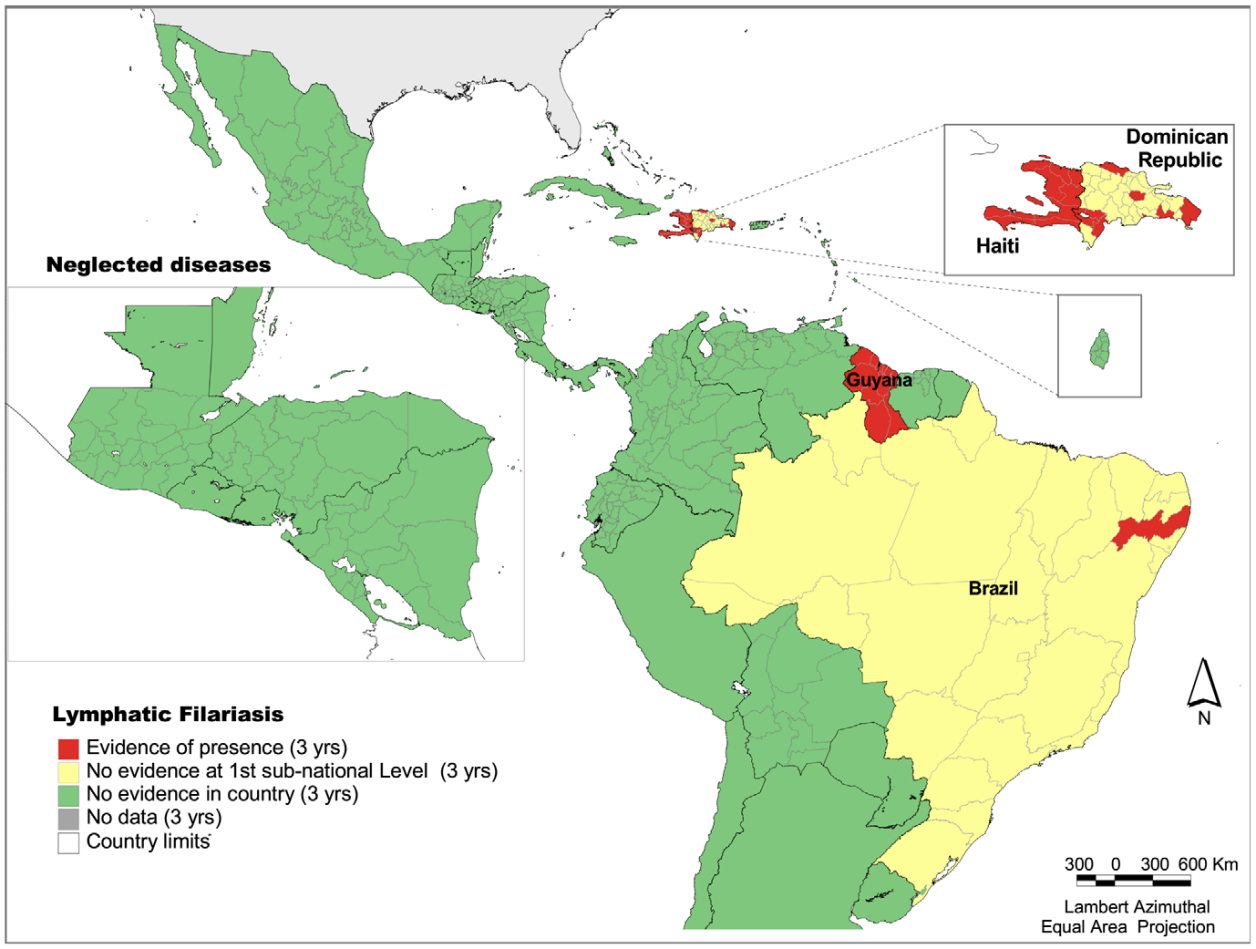
Presence of lymphatic filariasis at the first subnational level, Latin America and the Caribbean, 2005–2007. *Source:* PAHO based on reports submitted by the Ministries of Health to PAHO/WHO Program for the Elimination of Lymphatic Filariasis.

There is evidence of foci with active transmission of lymphatic filariasis in four countries: Brazil, Dominican Republic, Guyana, and Haiti. It is estimated that up to 11 million people are still at-risk of infection. The greatest population at-risk is in Haiti distributed among all first sub-national units. In Guyana, all first sub-national level units show evidence of the disease. In Brazil, there are foci in only one state (Pernambuco). There are 29 of approximately 570 administrative units at the first sub-national level that show evidence of lymphatic filariasis (Figure 1). Interventions are underway in each country.

### Onchocerciasis

**Figure 2:**
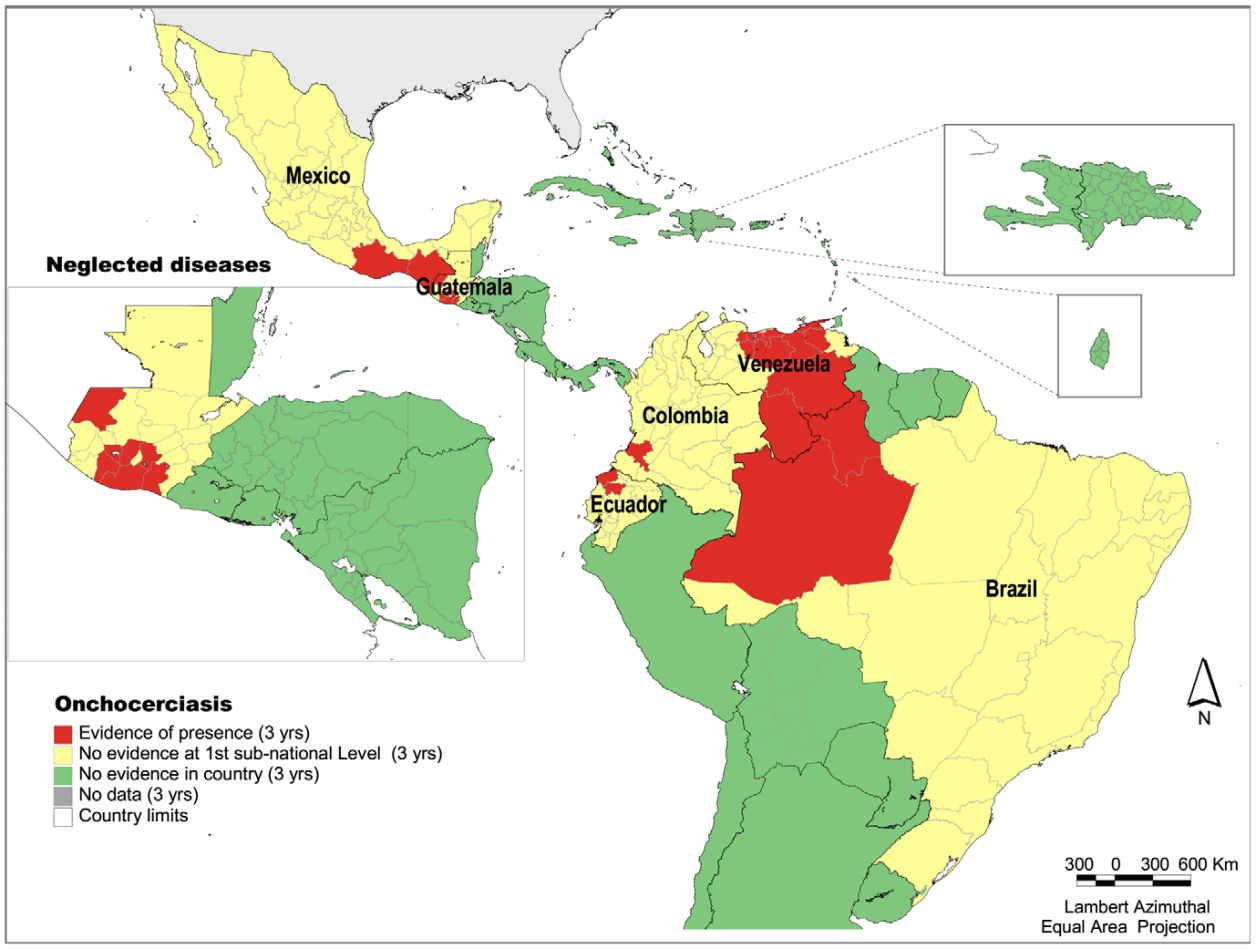
Presence of onchocerciasis at the first subnational level, Latin America and the Caribbean, 2005–2007. *Source:* ***PAHO*** based on data from: Onchocerciasis Elimination Program for the Americas (OEPA).

Of 570 administrative units at the first sub-national 25 showed evidence of the disease during the period covered by the study (Figure 2). Onchocerciasis is focalized in 13 foci in 6 countries: Brazil, Colombia, Ecuador, Guatemala, Mexico and Venezuela. It is estimated that approximately 500,000 people are at-risk in the Region. Most of these foci share state or international border. In Venezuela, foci extend along 11 states, with the population most at-risk living in remote communities. The disease greatly affects indigenous populations such as the Yanomami in the Amazon region of Brazil and Venezuela, the Mayas and other indigenous groups in Guatemala and Mexico, and those of African descent, who live in rural or mountainous areas [24].

By the end of 2007, transmission had been interrupted following sustained massive treatment at least twice a year in 6 foci, 1 in Colombia, 3 in Guatemala and 2 in Mexico. In order to certify elimination, these foci will be under surveillance for at least 3 years, which is the criterion set by the World Health Organization. There have been no new cases of blindness caused by onchocerciasis in the Region since 1995.

### Schistosomiasis

**Figure 3:**
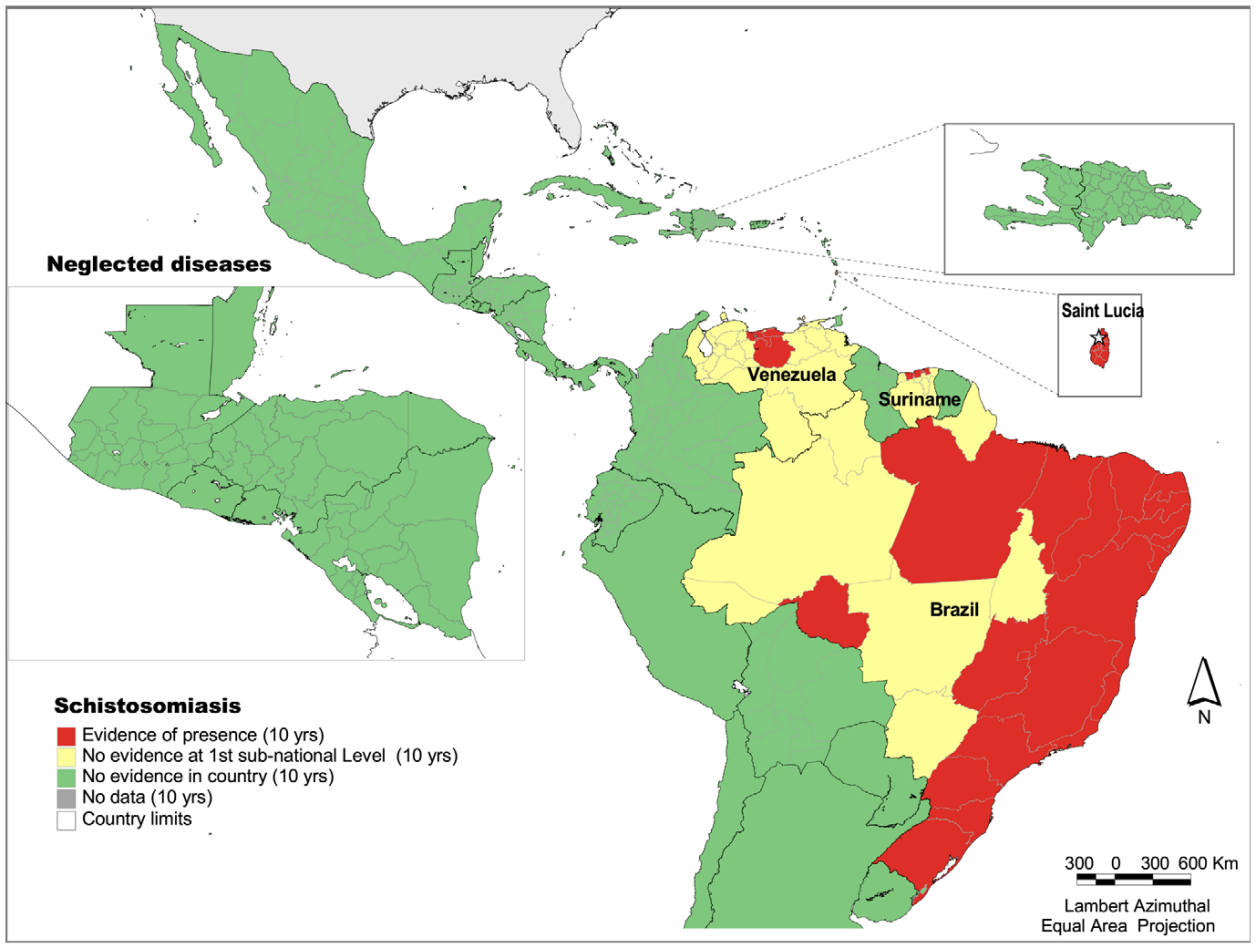
Presence of schistosomiasis at the first subnational level, Latin America and the Caribbean, 1998–2007. *Source:* PAHO based on several sources.

The disease is present in four countries in the Region: Brazil, St. Lucia, Suriname and Venezuela (Figure 3). A study suggests that the disease has been eliminated in previously endemic Martinique and Guadeloupe.^25^ In previous decades, the disease was known to exist in Puerto Rico and the Dominican Republic. However, no evidence of its presence has been found for the past 10 years, which suggests that an epidemiological study is needed to confirm elimination. Thirty nine of the 570 administrative units at the first sub-national level show presence of the disease. It is estimated that around 25 million people live at-risk in the Americas, and around 1 to 3 million people are estimated to be infected. In the Caribbean, the incidence of schistosomiasis has been dramatically reduced, making it possible for the disease to be eliminated (e.g. the French territories) [25]. The current status of schistosomiasis in Saint Lucia will be determined in a forthcoming planned study.

### Human rabies transmitted by dogs

**Figure 4:**
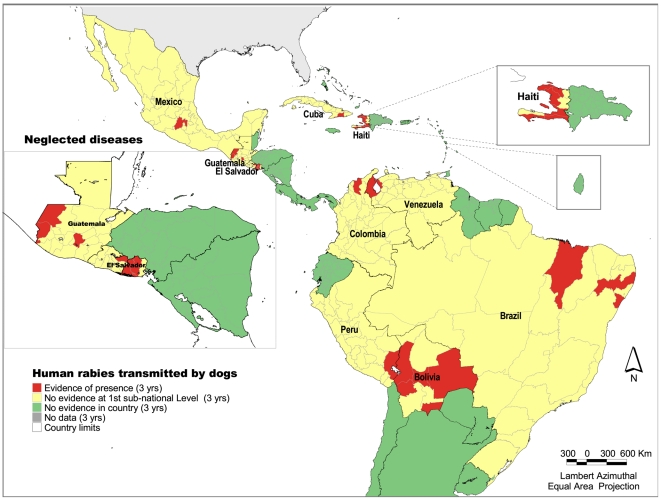
Presence of dog-transmitted human rabies cases at the first subnational level, Latin America and the Caribbean, 2005–2007. *Source:* PAHO based on SIRVERA Database, PAHO/PANAFTOSA.

One or more cases of human rabies transmitted by dogs have been reported in 10 countries: Bolivia, Brazil, Colombia, Cuba, El Salvador, Guatemala, Haiti, Mexico, Peru and Venezuela. Twenty administrative units of 570 present evidence of the disease (Figure 4). Around 60 millions people live in these states. The majority of the cases occur in the poor, outlying neighborhoods of large cities, mostly in Haiti and Bolivia [26]. Cases of human and canine rabies have been reduced by nearly 90% over the past 20 years since the inception of a Regional elimination program [27]. Even though the number of human cases is low due to country efforts (14 cases in 2007), the number of people who live in risk areas is still high because the virus circulation in the dog population.

### Trachoma

**Figure 5:**
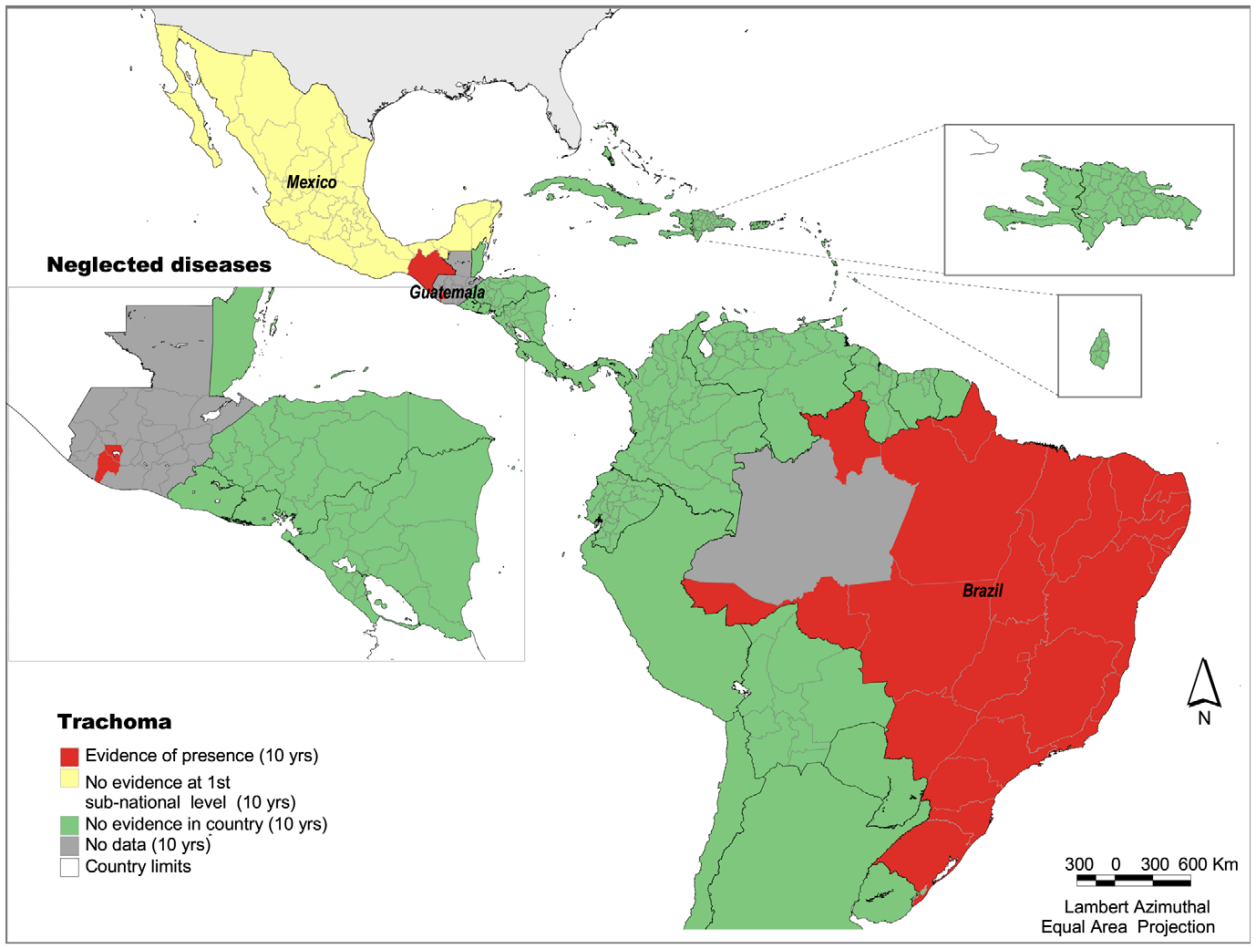
Presence of trachoma at the first subnational level, Latin America and the Caribbean, 1998–2007. *Source:* PAHO based on several sources.

There is evidence of the disease in three countries: Mexico, Guatemala and Brazil (Figure 5). Twenty-nine administrative units of 570 present evidence of the disease. It is estimated that around 50 million people live in risk areas and about 7,000 cases have been identified, most of them in Brazil. In Mexico, the disease is limited to 5 municipalities in one state (Chiapas) with low socioeconomic levels where it is primarily encountered in indigenous populations. In Guatemala, trachoma has been reported in 92 communities in two states. Due to the efforts of the Trachoma Prevention and Control Program, Chiapas, Mexico is close to eliminating the disease. Brazil has a national trachoma control program that is carrying out an active search for cases, and Guatemala is preparing a survey.

### Soil-transmitted helminths

**Figure 6:**
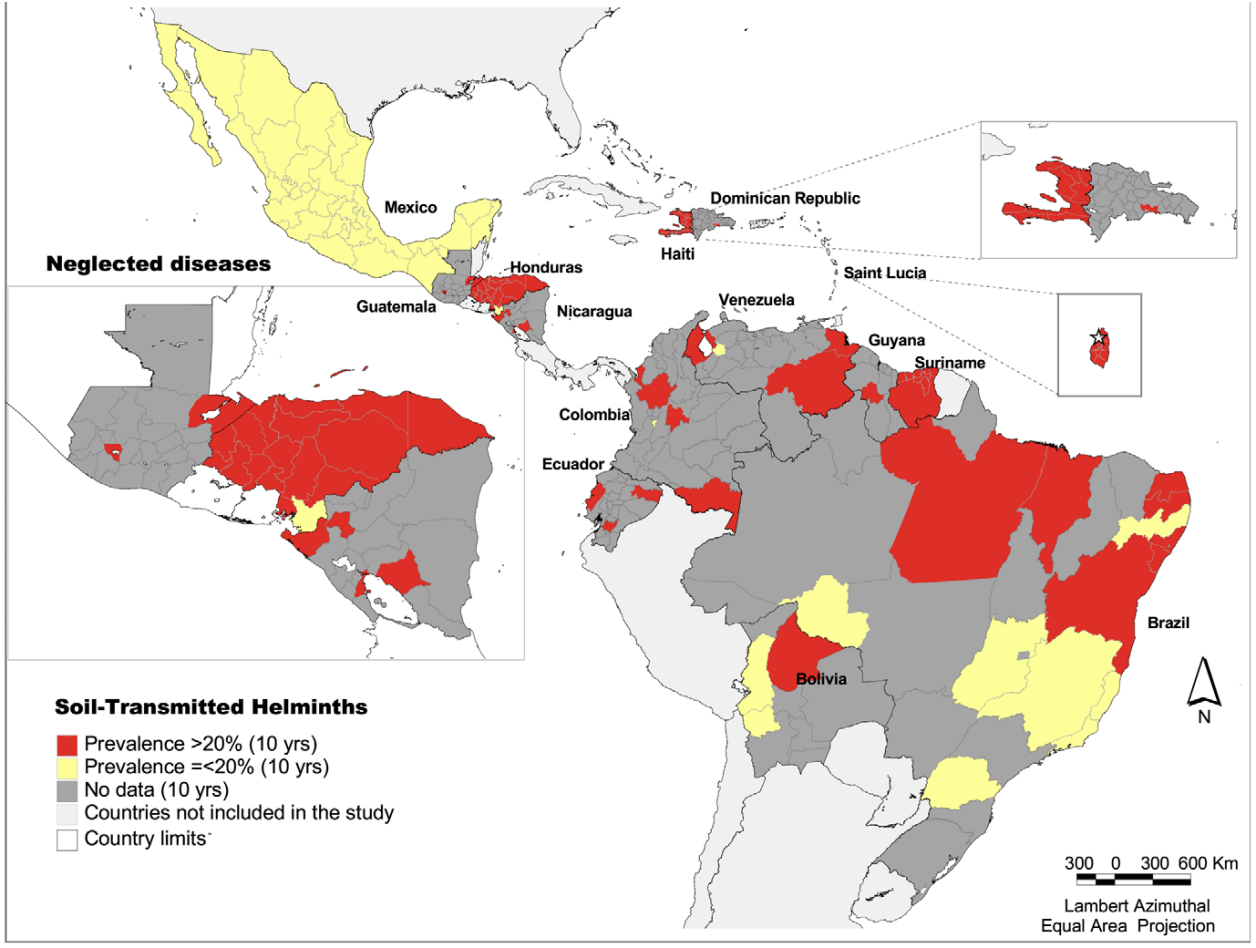
Prevalence of Soil-Transmitted Helminths according to existing studies, Latin America and the Caribbean, 1998–2007. *Source:* PAHO based on several sources.

Soil-transmitted helminths have been present in all the countries of the Region during the 10 year period covered by this study. Only 8 of 35 countries completed national level parasitological surveys (principally prevalence studies) for STH occurring between 2002 and 2006, with the following prevalence ranges at the first administrative level: Argentina 9.0–38.7%; Belize: 43.6–52.2%; Brazil: 2–36%, Haiti: 15–87%; Honduras: 12.2–97%, México: 0.01–16.3%, Nicaragua 27–80% and Venezuela: 3–19%. For the other countries included in this study, the following prevalence ranges were found according to a varying number of widely heterogenous point-prevalence studies: Bolivia: 4.5–65.4%, Colombia: 10.7–49.3%, Cuba: 4.5–47.3%, Dominican Republic: 5.3–55.3%; Ecuador: 28.5–71%, Guatemala: 12.7–68%, Guyana: 12.3–38%, Peru: 1.8–80.4%, Saint Lucia: 35–45%, Suriname: 36–43%. Figure 6 shows the mapping of STH in 14 countries in the Region with presence of at least one of the other five diseases included in the study [22], according to the available information. As shown in the map, important gaps of information on STH prevalence of infection at the first subnational level and below exist throughout the region.

### Overlapping diseases analysis

**Figure 7:**
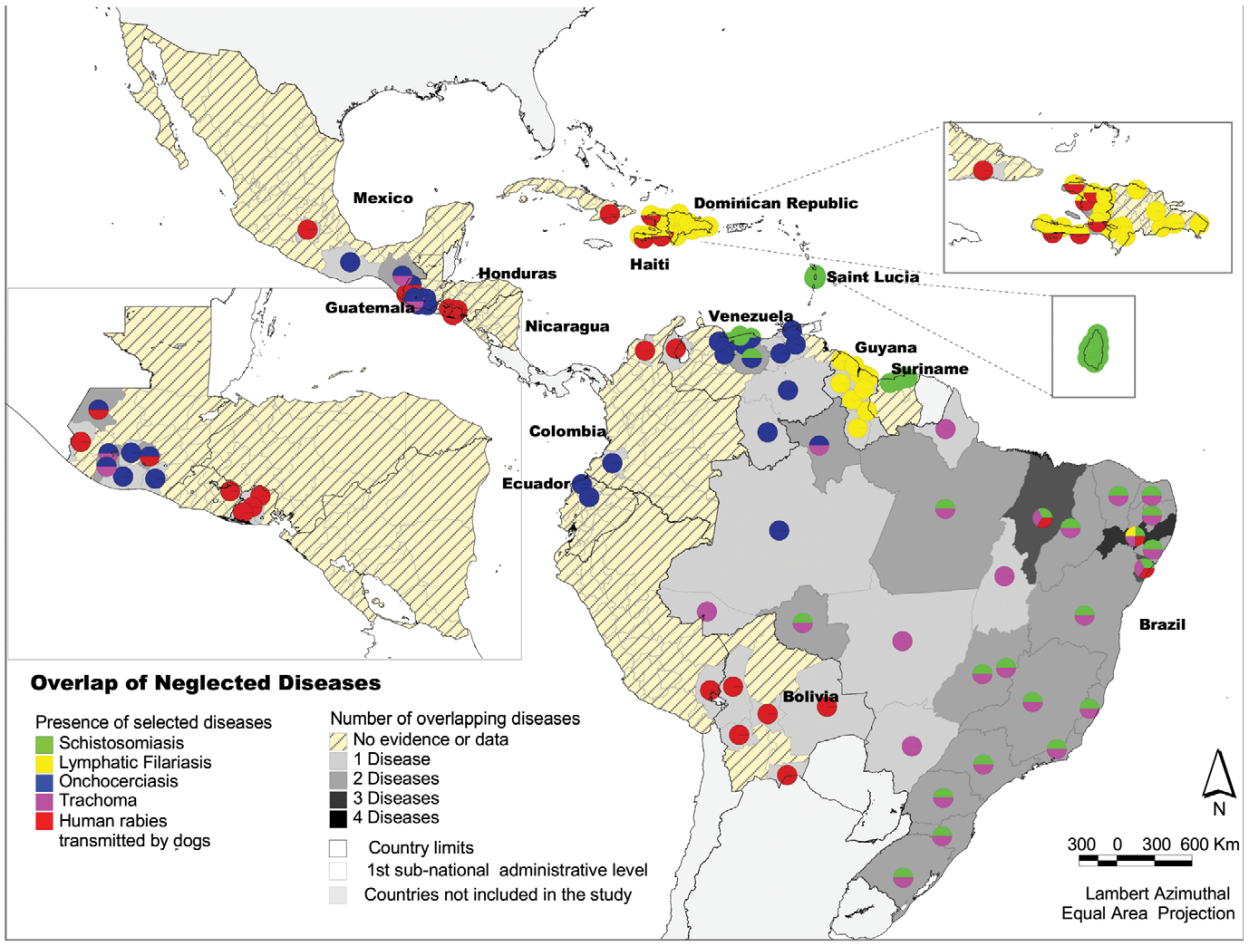
Overlapping diseases present in the country at the first subnational level, Latin America and the Caribbean. *Source:* PAHO, based on several sources.

There are 66 states with the presence of one of the selected diseases, 33 states with the presence of two of the diseases, two states present three of the five diseases and only one state has evidence of the presence of four of the five selected neglected diseases, classifying these overlapping areas as the “major hotspots” (Figure 7). The combination of diseases that overlap varies according to the country: for Brazil the greatest overlap is between trachoma and schistosomiasis; in Haiti it is lymphatic filariasis with human rabies transmitted by dogs; and in Venezuela it is schistosomiasis with onchocerciasis. While it is believed that soil-transmitted helminths are present, to a varying extent, in most of the first subnational administrative levels, STH were not included in the overlapping analysis given that this information is only available for some geopolitical units in selected countries.

## Discussion

This analysis suggests that the goal of elimination as a public health problem or drastic reduction of the selected neglected diseases is achievable in the Region. The focality of most of the neglected diseases and the countries' efforts, supported by international organizations and donors, present a positive scenario for combating neglected diseases in the Americas.

This is also the trend in the WHO South-East Asia Region, where conditions are now in place to eliminate some of the neglected diseases. The most important challenge will be continued good surveillance to determine whether these diseases remain in previously endemic areas and continued advocacy to sustain the political commitment [28].

Determined efforts are essential to advance towards the elimination of these poverty-related diseases and to reduce the social burden and inequality they produce. The fight against neglected diseases is one of the greatest challenges in achieving the Millennium Development Goals related to reducing infectious diseases (MDG 6) and reducing poverty (MDG 1). The recent increase in global donor support to fight neglected diseases and other poverty-related illnesses is expected to invigorate ongoing initiatives at the country level [29].

The study also suggests that most of these diseases could be considered part of the unfinished agenda for the Americas Region — diseases that have already been identified as priority targets for elimination and which, despite progress and the availability of cost-effective public health intervention measures (‘tool-ready” diseases), have lagged behind set goals. Some exceptions could be suggested for three of the five PAHO priority countries – defined as countries singled out for special attention due to the fact that their health status remains below regional averages [30]. In Haiti and Guyana, lymphatic filariasis is present in all first sub-national level units; in Bolivia and Haiti, rabies is present in almost half of the states. Besides soil-transmitted helminthiases, there was no evidence of any of the other five diseases studied in Honduras or Nicaragua, the other two PAHO priority countries. Further studies may be suggested in the priority countries to confirm that the selected diseases are not present.

In the area of work towards the elimination of onchocerciasis, it is important to mention the Onchocerciasis Elimination Program for the Americas (OEPA). This program is a multiagency and multinational, public-private partnership established in 1991 with the goal of eliminating all forms of clinical disease and interrupting disease transmission [31]. The strategy of OEPA has been, since its inception, to provide mass drug administration (MDA) to the eligible population in the Americas twice a year with a coverage rate of at least 85% in each treatment round during 10–12 years - the estimated life span of the adult parasite - complemented with health education, social mobilization and community participation. The program has been very successful, achieving by the end of 2009, the interruption of transmission in 7 of the 13 foci in the region, located in 4 countries, 3 in Guatemala, 2 in Mexico, and in each of the single foci in Colombia and Ecuador [32].

Schistosomiasis is another disease that requires additional surveys, particularly in the Caribbean, where there is currently only evidence of the presence of the disease in Saint Lucia and some coastal areas of Suriname. The study done in Martinique and Guadeloupe suggests that the disease has been eliminated from previously endemic areas in those territories [25]. However, there needs to be confirmation of the absence of schistosomiasis in other countries in the Caribbean, such as in the Dominican Republic, where the disease was formerly present and no studies were found from the past 10 years reporting the presence or absence of the disease.

Trachoma is present in almost all of the Brazilian states. Therefore, surveys of bordering areas in neighboring countries will be important in order to confirm that the disease is not present in other countries of the Region. Additionally, the countries' methodologies to measure trachoma could be reviewed to ensure that all are measuring the presence of the disease with the same criteria. For diseases with chronic symptoms, such as trachoma, prevalence is more difficult to measure, and the surveys that are required to estimate the burden of disease are often complex to conduct.

Human rabies, on the other hand, is a disease with easily identifiable symptoms. A regional notification system has been in place for several decades with information available on all countries. Human cases are reported by sub-national level, which helps to define the critical areas for intervention. In this case, any foci of human rabies transmitted by dogs are considered “hotspots” because the disease is no longer considered as a public health problem in the Americas [33]. However, even with the important reduction of human and canine cases, the achievement of the elimination goal will require continued support of major strategies such as human post-exposure prophylaxis, dog vaccination in the risk areas and surveillance.

The data on STH are very limited in that many of the available studies were at least 5 years old and most of them were circumscribed to very small geographic areas or to certain populations and their results could therefore not be extrapolated to other administrative levels. Thus, most of the countries in the Region are in need of mapping or re-mapping to complete and update the information on STH prevalence and report intensity of infection. Brazil will start in 2010 a national survey to update the epidemiological information on STH and schistosomiasis.

The overlapping analysis in this study indicates there are many sub-national units with more than one of the five selected diseases, suggesting that these areas could be considered “major hotspots” for integrated and intersectoral country approaches tackling the social determinants of health in addition to medical care of people with the diseases. Identifying hotspots at the first sub-national level is useful at the national and state political levels for advocacy purposes and for decision-makers to know the states where integrated approaches to tackle the neglected tropical diseases might be required and identify existing gaps and needs for information. However, elimination programs need maps refined to a lower level of disaggregation, down to the community level, for proper planning of integrated public health and intersectoral interventions. Therefore, further studies could be performed to identify “hotspots” to define more accurate baselines, possibly including additional diseases, socio-economic and environmental variables, and applying other clustering and correlation spatial analysis techniques. These types of studies could provide evidence for decision-making to establish integrated plans with a comprehensive approach and continue the ethical fight against neglected diseases.

In areas where overlapping of STH infections occurs either with lymphatic filariasis, onchocerciasis and schistosomiasis, there is an opportunity to integrate deworming for the two diseases. In fact, a deworming drug (albendazole) is included in the recommended combination for MDA used in the regional program to eliminate lymphatic filariasis. Besides deworming, another opportunity to integrate interventions to reduce the burden of several neglected tropical diseases is improving access to both safe water supply and adequate basic sanitation. This is critical to achieve a long-term reduction in both the burden (intensity of infection) and prevalence of STH in pre-school and school-age children, and also plays an important role in reducing the burden of schistosomiasis and lymphatic filariasis. In general in Latin America and the Caribbean, it is basic sanitation which lags behind. Thus regional estimates of the number of school-age children at risk of STH morbidity should be based on the proportion of this group without access to improved sanitation, that is, 15% of school-age children in urban areas and 55% in rural areas. In estimated numbers, this indicates that, for 2007, 12.9 million school-age children in urban areas and 13.4 million school-age children in rural areas, a total of 26.3 million school-age children in the LAC Region, were at high risk of infection by STH and therefore in need periodic deworming. According to PAHO's database on STH, the reported coverage reached in 2007 was 8.6 million presumably at-risk school-age children, or 32.7% of the estimated total population of school-age children at risk. Thus the treatment coverage gap was 17.7 million children (67.3% unreached). Looking at coverage targets based on the World Health Assembly Resolution 54.19 (2001) which set a benchmark of at least 75% to 100% treatment coverage targeted for school-age children at risk, our data indicate that coverage in the region should have reached at least 19.7 million and up to 26.3 million children in 2007.

It should be noted that there are some limitations to this study. One limitation is the potential for an underestimation of the occurrence of the disease in some countries, such as is the case for trachoma, information which is based on surveys. Additional studies need to be developed to confirm that this disease is not present in other areas. It is also worth pointing out that there is variance in the capacity that countries have to accurately represent the extent of diseases.

This analysis was conducted at the first subnational administrative level. This was done because this is the best level at which these diseases can be overlapped, given the available data. However, limitations exist when comparing first subnational level administrative units, given the considerable range of variation in terms of size and population of these units. Furthermore, in some cases the spatial presence of the diseases may be limited to foci that are much smaller than the entire state which has been marked as affected, as can be seen in the case of onchocerciasis. A similar limitation could be said of the information available on schistosomiasis in Southern as well as part of Southeastern Brazil, where the disease occurs but possibly at rates lower than the targets proposed in Table 2. Therefore, in some cases, this might lead to a visual overestimation of the dissemination of a disease. Also limitations exist when comparing first subnational level administrative units, given the considerable range of variation in terms of size and population of these units among the countries in the Region.

The possibility of overestimating the relevance of some diseases in countries with better surveillance systems as compared to other countries in the Region, mostly for diseases that need periodic surveys to be estimated, also exists.

It should be noted that while only five diseases were chosen for the overlapping study, other neglected diseases present throughout the Region – such as soil-transmitted helminthiasis, Chagas Diseases, congenital syphilis, and plague – must be considered once further epidemiological data is collected and incorporated into possible integrated approaches.

In the review of the literature, no other effort was found to have taken on the endeavor of mapping, for the entire region of Latin America and the Caribbean, these selected neglected diseases, as opposed to mapping one disease or country at a time.

The diseases highlighted in this study disproportionately affect vulnerable populations. In fact, several of the selected diseases – lymphatic filariasis, onchocerciasis, and schistosomiasis – were most likely imported to the Western Hemisphere through the slave trade and persist in the region as historical legacies of slavery [34]. The presence of these diseases can be considered as a social debt to the affected population and therefore, identified as priority areas for intervention.

Finally, the public health strategies that are used to eliminate or reduce diseases to acceptable levels go beyond routine control measures. In order to strengthen the efforts against diseases related to poverty as a group, countries and territories in the Region could develop integrated plans under the same framework. Plans and guidelines are already available to eliminate or control the selected diseases, as are tools such as drugs and diagnostic techniques to support surveillance systems. For these diseases to be eliminated, emphasis should be placed at the country level on evidence-based decisions for strengthening health surveillance systems, mapping the diseases to identify remaining “hotspots,” and identification of geopolitical areas in which the diseases overlap (“major hotspots”). It is necessary to ensure that resources are available for the primary care system to help reduce inequalities in the fight against these diseases. Inter-programmatic interventions should be established that integrate the various plans, breaking the barrier between diseases and disciplines. Pursuing community participation and intersectoral partnerships is crucial, as involving the community, stakeholders and all potential partners within and outside the health sector will make actions sustainable [18].

## Supporting Information

Alternative Language Abstract S1 Abstract translated to Spanish and Portuguese.
(0.03 MB DOC)
