# Supplementary material for: Elimination of Neglected Diseases in Latin America and the Caribbean: A Mapping of Selected Diseases
Source: PLoS Negl Trop Dis. 2011 Feb 15;5(2):e964. doi: 10.1371/journal.pntd.0000964 (PMC3039687; doi:10.1371/journal.pntd.0000964)
Supplement: Alternative Language Abstract S1 — Abstract translated to Spanish and Portuguese. (0.03 MB DOC) [file pntd.0000964.s001.doc]

**Translation of abstracts into Spanish and Portuguese**

**Translation of abstract into Spanish by RS Nicholls**

En América Latina y el Caribe, aproximadamente 195 millones de personas viven en la pobreza, situación que aumenta la carga de algunas enfermedades infecciosas. Las enfermedades desatendidas, en particular, a menudo afectan a los sectores más pobres y marginados de la población. Existen herramientas para combatir estas enfermedades, por lo cual es imperativo trabajar hacia su eliminación. En 2009, la Organización Panamericana de la Salud (OPS) recibió el mandato de apoyar a los países de la región para lograr la eliminación de las enfermedades desatendidas y de otras enfermedades infecciosas relacionadas con la pobreza. El objetivo de este estudio es analizar, mediante técnicas de geo-procesamiento, la presencia de algunas enfermedades seleccionadas. Cinco enfermedades con información disponible a primer nivel subnacional (estados) fueron mapeadas, mostrando la presencia de enfermedades ("áreas críticas") y su superposición ("principales áreas críticas"). En los 45 países o territorios (aproximadamente 570 estados) de la Región, se encuentran: la filariasis linfática en cuatro países (29 estados), la oncocercosis en seis países (25 estados), la esquistosomiasis en cuatro países (39 estados), el tracoma en tres países (29 estados), y la rabia humana transmitida por perros en diez países (20 estados). De los 108 estados con una o más de las enfermedades seleccionadas, 36 estados presentan las enfermedades en áreas de superposición ("principales áreas críticas"). Se incluyó información adicional acerca de los geohelmintos. El análisis sugiere que la mayoría de las enfermedades seleccionadas no están ampliamente diseminadas y pueden considerarse como parte de una agenda inconclusa con el objetivo de eliminarlas. Se requiere de planes con un enfoque integral que garanticen el acceso a métodos de diagnóstico y tratamiento, así como el establecimiento de una agenda multisectorial que aborde los determinantes sociales, incluyendo el acceso a agua potable y a condiciones adecuadas de saneamiento básico. Los estudios futuros pueden incluir enfermedades adicionales y variables socio-económicas y ambientales.

Listen

Read phonetically

Dictionary - [View detailed dictionary](http://www.google.com/dictionary?source=translation&hl=en&q=In Latin America and the Caribbean, around 195 million people live in poverty, a situation that increases the burden of some infectious diseases. Neglected diseases, in particular, are often restricted to poor, marginalized sections of the population. Tools exist to combat these diseases, making it imperative to work towards their elimination. In 2009, the Pan American Health Organization (PAHO) received a mandate to support the countries in the Region in eliminating neglected diseases and other poverty-related infections. The objective of this study is to analyze the presence of selected diseases using geo-processing techniques. Five diseases with information available at the first sub-national level (states) were mapped, showing the presence of the disease (“hotspots)

**Translation of abstract into Portuguese by MC Schneider**

Na América Latina e Caribe, aproximadamente 195 milhões de pessoas vivem na pobreza, situação que aumenta a carga de algumas doenças infecciosas. Em particular, as doenças desatendidas afetam frequentemente os setores mais pobres e marginalizados da população. Existem ferramentas para combater estas doenças, razão pela qual é imperativo trabalhar para sua eliminação. Em 2009, a Organização Pan-Americana da Saúde (OPAS) recebeu o mandato de apoiar os países da Região para conseguir a eliminação das doenças desatendidas e de outras doenças infecciosas relacionadas com a pobreza. O objetivo deste estudo é analisar, mediante técnicas de geoprocessamento, a presença de algumas doenças selecionadas. Cinco doenças com informação disponível em primeiro o nível subnacional (estados) foram mapeadas, mostrando a presença de doenças ("áreas críticas") e sua sobreposição ("principais áreas críticas"). Nos 45 países ou territórios (aproximadamente 570 estados) da Região, encontram-se: filariose linfática em quatro países (29 estados), oncocercose em seis países (25 estados), esquistossomíasse em quatro países (39 estados), tracoma em três países (29 estados), e raiva humana transmitida por cães em dez países (20 estados). Dos 108 estados com uma ou mais das doenças selecionadas, 36 estados apresentam as doenças em áreas de sobreposição ("principais áreas críticas"). Foi incluía informação adicional em relação a helmintíase transmitida pelo solo. A análise sugere que a maioria das doenças selecionadas não estão amplamente disseminadas e podem ser consideradas como parte de uma agenda inconclusa com o objetivo de eliminá-las. São requeridos planos com um enfoque integral que garantam o acesso a métodos de diagnóstico e tratamento, bem como do estabelecimento de uma agenda multisetorial que aborde os determinantes sociais, incluindo o acesso a água potável e a condições adequadas de saneamento básico. Em estudos futuros podem ser incluidas doenças adicionais e variáveis socioeconômicas e ambientais.
